# Verification of Geometric Model-Based Plant Phenotyping Methods for Studies of Xerophytic Plants

**DOI:** 10.3390/s16070924

**Published:** 2016-06-27

**Authors:** Paweł Drapikowski, Ewa Kazimierczak-Grygiel, Dominik Korecki, Justyna Wiland-Szymańska

**Affiliations:** 1 Institute of Control and Information Engineering, Faculty of Electrical Engineering, Poznan University of Technology, Piotrowo 3a, 60-965 Poznan, Poland; korecki.dominik@gmail.com; 2 Botanical Garden, Adam Mickiewicz University, Dąbrowskiego 165, 60-594 Poznan, Poland; ewakg@amu.edu.pl (E.K.-J.); wiland@amu.edu.pl (J.W.-S.); 3 Department of Plant Taxonomy, Faculty of Biology, Adam Mickiewicz University, Umultowska 89, 61-614 Poznan, Poland

**Keywords:** plant digitalization, morphological parameters, DAVID laser scanning system

## Abstract

This paper presents the results of verification of certain non-contact measurement methods of plant scanning to estimate morphological parameters such as length, width, area, volume of leaves and/or stems on the basis of computer models. The best results in reproducing the shape of scanned objects up to 50 cm in height were obtained with the structured-light DAVID Laserscanner. The optimal triangle mesh resolution for scanned surfaces was determined with the measurement error taken into account. The research suggests that measuring morphological parameters from computer models can supplement or even replace phenotyping with classic methods. Calculating precise values of area and volume makes determination of the S/V (surface/volume) ratio for cacti and other succulents possible, whereas for classic methods the result is an approximation only. In addition, the possibility of scanning and measuring plant species which differ in morphology was investigated.

## 1. Introduction

Plant phenotyping is the comprehensive assessment of complex plant traits including their architecture. Classical methods are used to study variability, adaptation to environmental conditions and pace of growth during the vegetation period. So far, plant phenotyping has been mainly limited to crop plants or the model plant *Arabidopsis thaliana* (L.) Heynh [1]. In many cases, classical biometric measurement methods are sufficient. However, there are plants whose complex spatial structure and susceptibility to damage make employing conventional measurement techniques impossible or exceedingly difficult. Therefore, there is a need for new platforms and solutions that enable phenotypic evaluation of such species. In such cases, non-contact measurement techniques need to be used: preparation of a three-dimensional (3D) computer model (digitalization) and capturing plant traits based on it [1,2].

One group of plants that has not been used as an object for 3D phenotyping is xerophytes. Representatives of this remarkable group are interesting for several reasons. These plants show various morphological, anatomical and physiological adaptations to living in dry conditions and in sustaining an intensive herbivory [3,4,5,6,7]. They constitute the main vegetation in some desert and semi-desert environments and are therefore a subject of intensive ecological study [8]. Their medicinal properties are recognized by both traditional and contemporary medicine as well as veterinary science [9,10,11,12].

Xerophytes are also desirable ornamental plants worldwide. The demand for these plants is so high that their existence in the wild is threatened [13]. Therefore, many xerophytes, including the whole cacti family and genera *Aloe* L. and *Pachypodium* Lindl [14,15], are protected by international law under the Washington Convention (CITES) as well as by national laws. Therefore, customs officers must be able to recognize them properly to prevent illegal traffic. The diversity of these plants is, however, so great that only specialists are able to recognize them and classify them accordingly. Comprehensive virtual guides that would enable rapid and simple identification of these plants do not exist.

Climate change as well population growth have created an urgent need for sustainable high-yield crop production. Phenotypic studies of plants adapted to difficult environmental conditions can be helpful in finding new ways for crop improvement [16].

**Figure 1:**
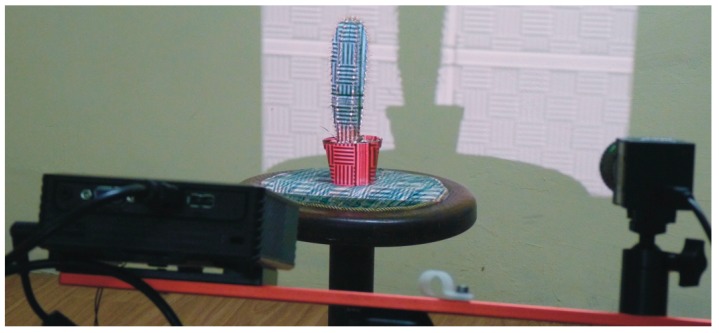
Scanning a succulent plant in the pot with the structured light-based DAVID Laserscanner [17].

Various types of scanners make non-contact plant digitalization possible. Most often they work by casting structured light in various patterns onto the object and recording the images (Figure 1) [17]. On the basis of disruptions in the pattern caused by the object’s surface, a three-dimensional point cloud is determined, which is further triangulated into the form of a triangle mesh. The triangle mesh computer model is then processed to determine morphological parameters of interest.

Non-contact measurements of selected characteristics are possible using the Ubiquitous Sensor Network proposed by Suk et al. [18]. An example of image-based plant phenotyping on a much larger scale was presented by Kircherer et al. [19], and numerous additional examples can be found [1,2,20,21,22,23].

Scanning larger objects is feasible with the use of, for instance, a Kinect scanner [24]. Recently, the photogrammetric method for creating computer models has been gaining popularity. It uses a previously captured set of images. There are no limits to the size of objects: the only restriction is the ability to take a photograph. Professional photographic equipment is not required, only a digital camera is necessary [25]. These scanning methods require relatively low-cost devices which offer acceptable metrological characteristics. Extensive metrological and usability descriptions of those devices for plant measurement have been published [26,27]. Better results in scanning plants of very complex shapes have been achieved with hand-held scanners, e.g., Artec S [28] or HandyScan EXASCAN [29]. Also, triangulation scanners (typically more accurate), mounted on special measuring arms [30,31], can be used for this purpose, e.g., Artec S and Perceptron ScanWorks V5 with the Romer Infinite 2.0 arm. However, their prices are far higher than those of hand-held devices.

One frequently measured parameter in plant biology is S/V (surface to volume ratio). It is easy to determine it for a flat-leaved plant when the surface and thickness of a leaf are measured. It is also possible to measure *in situ* with a portable scanner, e.g., Portable Living Leaf Area Meter [32]. For geometrically more complex plants, determination of S/V requires calculation of the surface and volume.

The present work verifies certain non-contact measurement methods of certain plant morphological parameters (length, width, area and volume of leaf) that are otherwise very difficult or impossible to determine with traditional techniques.

The paper also describes difficulties in plant scanning due to complicated surfaces, the presence of thorns or prickles that diffuse light, as well as hard-to-reach fragments that make it impossible to recreate the surface.

## 2. Materials and Methods

### 2.1. Materials

The research was conducted on specimens belonging to nine species of xerophytic plants grown in the Botanical Garden of the Adam Mickiewicz University in Poznan, Poland. The species are representatives of various ecological groups of xerophytes. They are all included in the CITES Appendices (Table 1). The families and names of species were applied according to the TROPICOS database [33].

### 2.2. Methods

#### 2.2.1. Distance Measurement between Two Points

**Figure 2:**
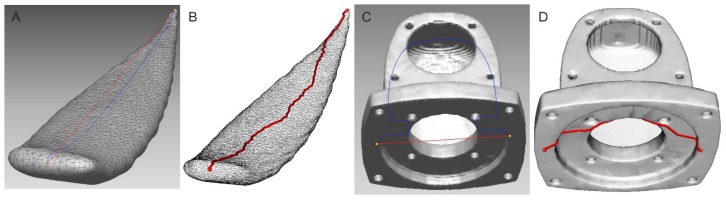
Finding the shortest path between points (part of an *Aloe marlothii* leaf): (**A**) A line projection onto the surface; (**B**) Triangle mesh-based; (**C**) An example of the incorrect shortest path; (**D**) The correct triangle mesh-based path.

A three-dimensional triangular mesh forms a computer model. There are two ways to measure the distance between two given points in such a model. The first is to run the shortest route between the given points along the edges of triangles forming the mesh. The mesh is transformed to a graph which has the distances as the weights. Dijkstra’s algorithm was used to find the shortest route in the graph (Figure 2B) [34]. Dijkstra’s algorithm finds the shortest paths from the source node to all other nodes in the graph, producing a shortest-path tree. The algorithm stops when the destination node is determined.

The second method is projection of a straight line which joins two points onto the surface of the object and in finding the shortest path outside the edges of the triangles (Figure 2A). This latter method is more accurate, as it shows indeed the shortest route, but it is more difficult to implement and does not always yield the correct result. This technique is used by specialized 3D RapidForm 2006 software to process the scanned data [35]. Even so, when the surface is a complex geometric shape, the distance is either not calculated at all or is calculated incorrectly (blue line in Figure 2C). An object depicted in Figure 2C is only an example of an improper shortest-path estimation by commercially available software [35]. Finding the shortest path with Dijkstra’s method for the same object is correct and shown in Figure 2D. Red lines in Figure 2A,C depict the direct connection between points but the blue ones depict the real connection based on mesh.

#### 2.2.2. Leaf’s Length Estimation as a Center Line

**Figure 3:**
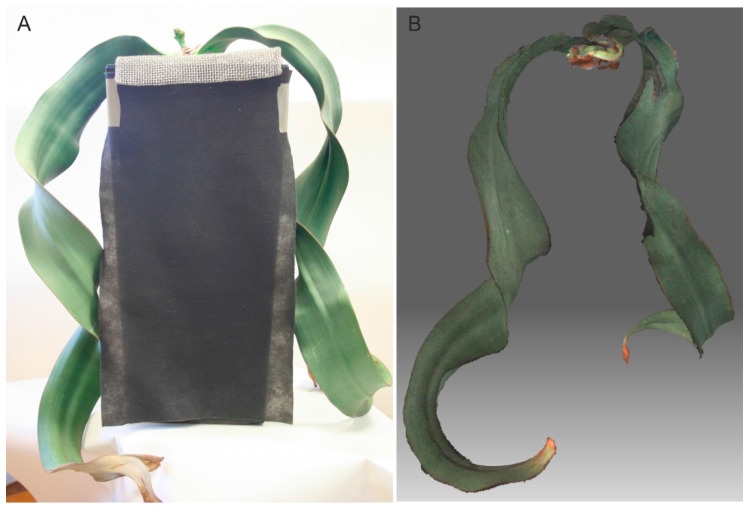
*Welwitschia mirabilis*: (**A**) Natural picture; (**B**) Computer model.

*Welwitschia mirabilis* is among the species that are difficult to measure with classic methods because of the shape of the leaves and susceptibility to damage (Figure 3A).

**Figure 4:**
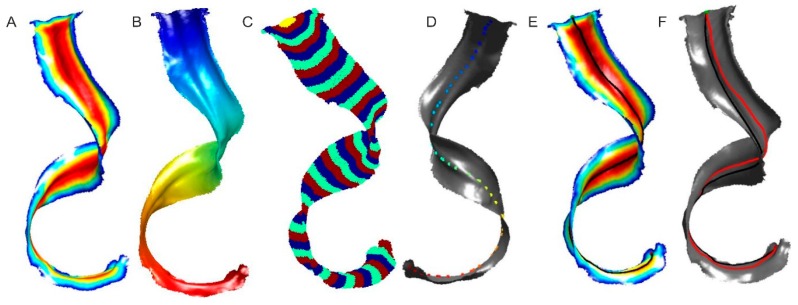
Steps to determine the length of a leaf: (**A**) Map of distances from the edge of the leaf; (**B**) Map of distances from the starting point to the endpoint; (**C**) Area division; (**D**) Vertices furthest away from the edge; (**E**) The shortest path; (**F**) Comparison of the path through the middle of the leaf (red line) and the shortest path in the geometric sense (black line) [37].

The length of the leaf is usually measured along the line going through its middle. When the leaf is twisted, as is the case with *Welwitschia mirabilis* (Figure 3), Dijkstra’s algorithm does not give good results because the shortest path does not lie in the middle of the leaf. To determine the length of the leaf, multiple steps are needed:

- finding the outer edges of the leaf,
- mapping the distances from all vertices to the edges in the model using the Fast Marching Method (FMM) [36] (Figure 4A),
- mapping the distances between all vertices in the triangular mesh from the starting point using the FMM method and division into a given number of areas (Figure 4B,C),
- determining the vertices that are furthest from the object’s edges in every area except the one containing the starting point and the endpoint (Figure 4D),
- determining the shortest routes between vertices set in the previous step, taking into account the starting point and the endpoint, then summing them (Figure 4E).

Figure 4F shows the paths obtained with both methods between the starting point and the ending point. Red presents the line put through the middle of the leaf while black presents the shortest geometric path between those points determined with Dijkstra’s method. It works well when the object is not geometrically complex, for instance with *Aloe marlothii* leaves. However, with objects such as leaves of *Welwitschia mirabilis*, it becomes necessary to use the multi-step procedure detailed above.

#### 2.2.3. Errors of Measurement Based on Computer Models

The use of computer models requires verification of precision in their creation and in the accuracy of measurements. It is assumed that the precision of models is based on manufacturer data which for the DAVID 3D SLA-1 scanner is 0.1% of the object size. Such precision is sufficient for measurements of plants’ characteristics. Measurement accuracy based on triangle mesh models also depends on mesh resolution. Error of measurement of different characteristics depending on mesh resolution is calculated in relation to the densest mesh model taken as the reference (*Model*_7_, Equation (1)).

(1) $$Error=\frac{Model_{7}-Model_{n}}{Model_{7}}\cdot 100\%\;where\;n\in \langle 1,6\rangle$$

Detailed analysis and results are presented in Section 3.1.

## 3. Results

### 3.1. Errors in Measurement of Distances, Areas and Volumes on the Basis of Computer Models

**Figure 5:**
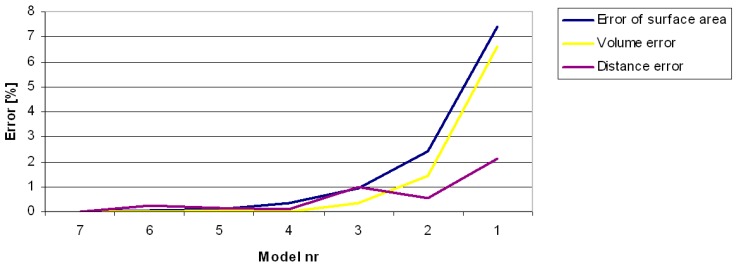
Relation between model resolution and measurement errors.

**Figure 6:**
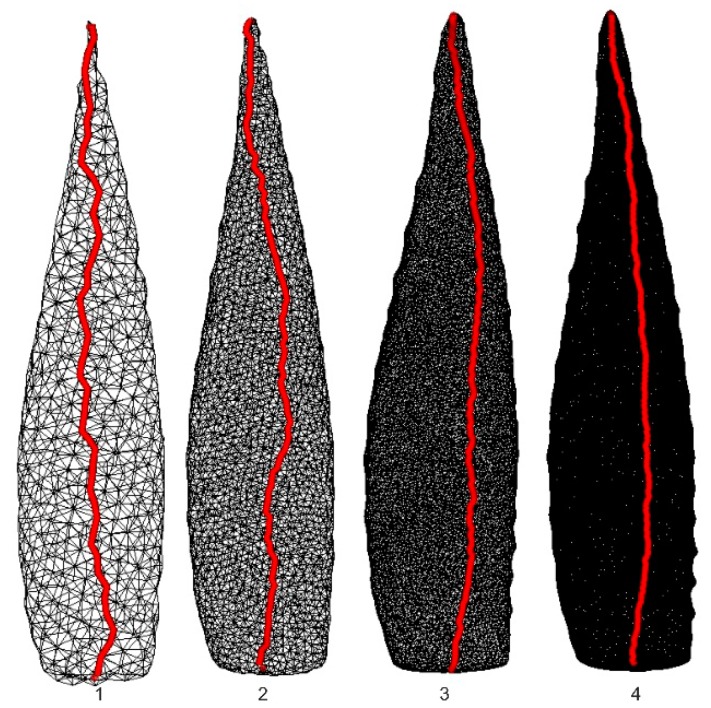
Fragments of the same object (an *Aloe marlothii* leaf) depicted as the triangular mesh with different resolutions and with the shortest distances between two points.

Errors in determining the shortest distance between the points, the area or the volume in the triangular mesh model depend on the spatial resolution of the mesh. Spatial resolution is set in the final stage of model creation in the scanning software. In order to analyze how the mesh resolution influences measurement accuracy, the same model of the *Aloe marlothii* leaf was processed with different resolutions understood as the mean distance between the points. Table 2 presents the results of measuring the distance, area, and volume. The number of points and triangles indicates that Model 3 is approximately 60 times “smaller” (in the mean of the number of points and triangles) than Model 7 but its error is only 1% with respect to Model 7. Figure 5 depicts the relationship of measurement errors to the reference model. A graphic representation is shown in Figure 6, where the red line depicts the distance between points according to Dijkstra’s algorithm. Models 5, 6, and 7 are not shown because the mesh density is such that the triangle mesh is not visible.

On the basis of the diagram shown in Figure 5, it can be said that a model with the triangular mesh resolution of 1 mm or less yields errors lower than 1%. Such accuracy is sufficient for measuring morphological properties of plants when using computer models. Spatial triangular mesh resolution is a parameter of final-stage modeling in the data-processing pipeline. Applying very fine mesh resolutions results in a vast number of triangles, which lengthens the processing time and, in extreme cases, makes calculations impossible due to the limited memory available, especially in 32 bit software. In Dijkstra’s method, the entire triangular mesh dataset is converted to a graph, which requires corresponding memory allocation. Proper mesh resolution is required for accurate measurements.

The penultimate row in Table 2 lists errors in length calculation between the points, determined with the mesh and the shortest geometric value (blue line shown in Figure 2A). This result was taken as the reference measurement. It can be seen that for models 3 to 7 the error is 3% to 5%. For a distance of 180 mm it gives about 7 mm. This error can be treated as the method error, stemming from measurements based on models with triangular mesh as the surface.

In phenotyping, it is more important to determine relative values of parameters (growth changes) than their absolute values. In that case, the systematic error resulting from the approximate distance calculation will not play a role. Moreover the S/V ratio, frequently calculated in plant science, remains unchanged regardless of the triangular mesh resolution (Table 2). The general assumption in volume calculation is that the model is of closed surface.

Measurement accuracy depends on an accurate model and on the measurement procedure. Paulus et al. [26] performed a comprehensive investigation of model accuracy using the DAVID 3D Laserscanner. To enhance this study, a sphere with a radius of 50 mm was used to validate the calculation of volume and surface area. The sphere was triangulated with an angular resolution of 2° which gives a distance between vertices of 1.7 mm on the equator. The measured volume and surface area were 523,329 mm^3^ and 31,408 mm^2^, which compare well with the calculated volume and surface area of 523,598 mm^3^ and 31,415 mm^2^. Thus, the triangulation has negligible error.

### 3.2. DAVID Laserscanner Scanning Results and Data Processing

For specimens smaller than 600 mm, the best device for data acquisition is a structured-light scanner. The authors used the DAVID 3D SLA-1 scanner [17] in their research. A complete model of the plant was obtained by combining 30 partial scans. The Iterative Closest Point (ICP) method was used to combine them. Data acquisition and scan combination were performed with the use of the manufacturer’s software. Figure 3 shows the image of the plant (*Welwitschia mirabilis*) and its geometric model with the texture visible. After the model in the form of a triangle mesh is obtained, software developed by the authors was used for the calculation of morphological parameters. The software was developed based on the Visualization Toolkit (VTK) library [38,39] commonly used in the computer graphics community. For twisted leaf length estimations such as those for *Welwitschia mirabilis* leafs a spatial software tool was developed based on the Matlab environment [37].

### 3.3. Hand-Held and Kinect Scanning Results

**Figure 7:**
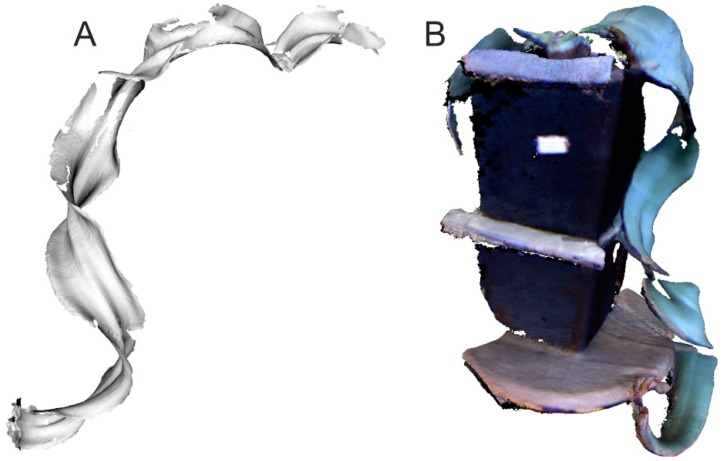
Model of a *Welwitschia mirabilis* leaf obtained with: (**A**) A hand-held and (**B**) Kinect scanner.

Owing to plant size limitations when using the structured-light DAVID Laserscanner, the feasibility of working with a hand-held and Kinect scanner was assessed using the *Welwitschia mirabilis* plant. The device HandyScan EXASCAN produced by Creaform was used [29]. This somewhat older scanner (made in 2009) requires the placement of markers onto the object or its surroundings to aid in positioning the scan head. Current hand-held scanners no longer have this disadvantage and the positioning is performed based on the scanned point cloud. The leaf models are shown in Figure 7. The appearance is much worse than for the models obtained with the DAVID Laserscanner (Figure 3), as seen in the number of missing fragments.

### 3.4. Geometric Models of Large-Size Plants

**Figure 8:**
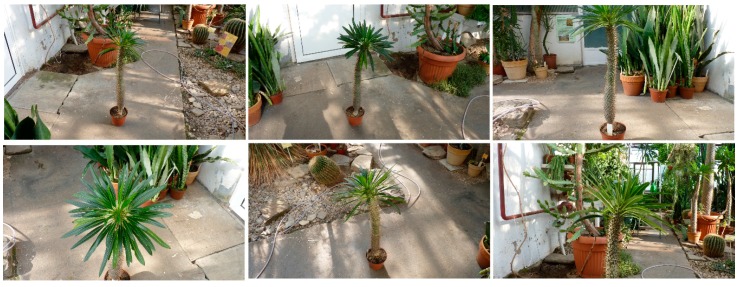
Several sample images from the *Pachypodium lamerei* series.

**Figure 9:**
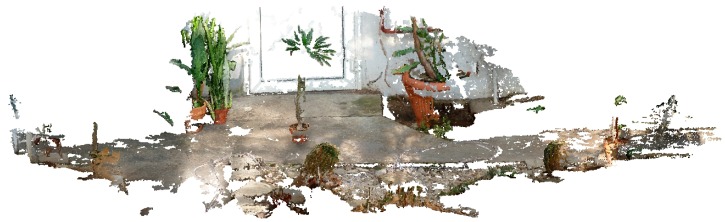
Scene reconstructed from the images with a point cloud and a visible texture.

**Figure 10:**
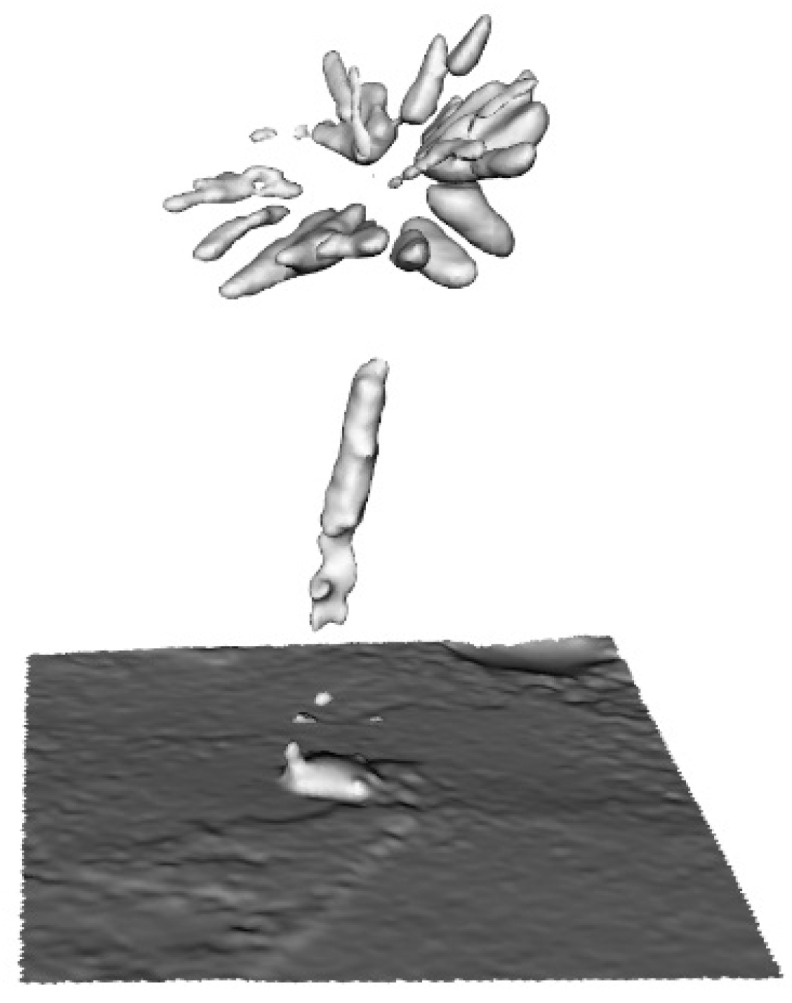
Model of *Pachypodium lamerei*.

Twenty-nine images of a specimen of *Pachypodium lamerei*, photographed with the digital camera Panasonic Lumix DMC-FZ38 in the glasshouse environment, are presented in the Figure 8. Image processing and object reconstruction were done with the help of VisualSFM software [40,41] and the reconstruction effect can be seen in Figure 9 and Figure 10.

The point cloud presented in Figure 9 was limited to the area which contains the object to be reconstructed. The reconstruction was performed with the Poisson method [42]. The main challenge in surface reconstruction from a point cloud in photogrammetry is to fit the surface according to the real object shape in the presence of noise, holes and a sparse point cloud. In the Poisson method, because t uses an indicator function defined as 1 for points inside the model and as 0 for points outside, the reconstructed surface is an extracted isosurface. This result is usually better than that of a Delaunay triangulation.

Clearly, the model shown in Figure 10 is not suitable for species identification, let alone phenotyping. A relatively small number of images influenced the result. To overcome the problem of large plant digitalization a multi-camera setup as proposed by Nguyen et al. [43] is recommended.

### 3.5. Detecting the Effect of Different Kinds of Surfaces on Effective Plant Scanning

**Figure 11:**
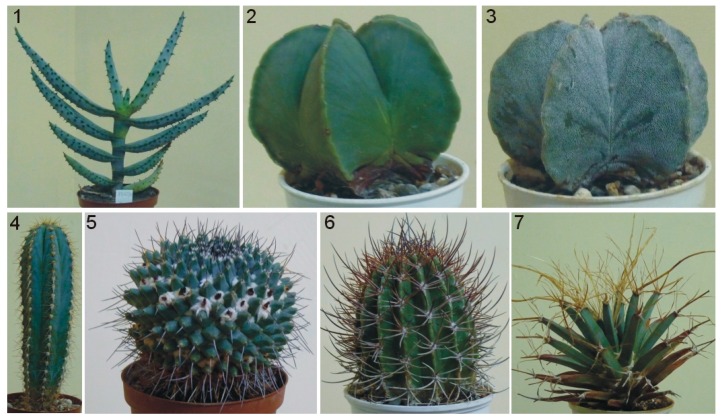
Scanned plant species which had computer models created. 1. Aloe marlothii; 2. Astrophytum capricorne; 3. Astrophytum myriostigma; 4. Pilosocereus pachycladus; 5. Mammillaria magnimamma; 6. Echinopsis leucantha; 7. Leuchtenbergia principis.

**Figure 12:**
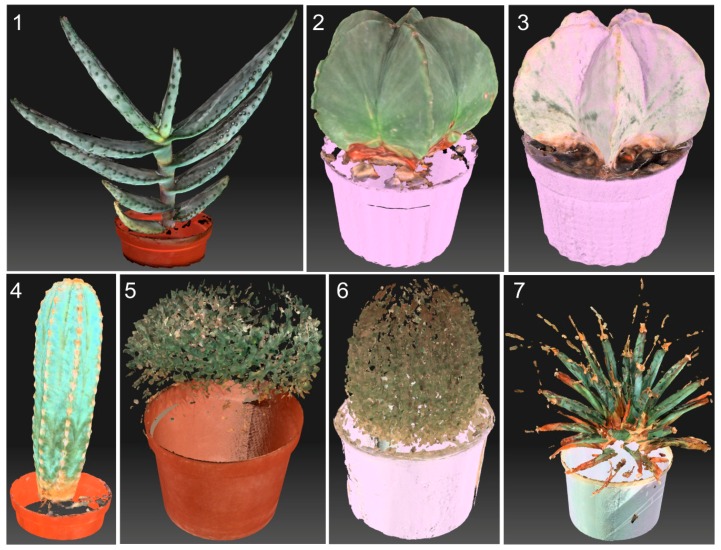
Computer models for plants in Figure 11.

The large diversity of plants as regards their shape and type of surface is sometimes not conducive to obtaining scan-based computer models that serve well for phenotyping. In order to assess which surface feature influences this problem, seven plant species were scanned (Figure 11) to obtain their computer models (Figure 12). The models were obtained with the DAVID 3D Laserscanner.

It can be claimed that computer models of plants similar to numbers 1 to 4 are usable for measurement purposes. Plants number 5 to 7 are not suitable for scanning with structured light devices due to the presence of thorns and/or prickles which disperse the light. Such computer models are incomplete and not fit for measurement purposes. Such plants can be scanned using a triangulation sensor, e.g., Perceptron Scanworks, coupled to a Romer arm. This arrangement allows maximum width (140 mm) and depth of field (110 mm), with a minimum point-to-point distance of 12 micrometers. A measurement arm or robotic scanner can move around the plant to capture data that would be obscured by single-angle measurements. The scanner price, reportedly $100,000, is, however, a drawback.

Before measurements are taken, the model must be corrected to eliminate features not relevant to the study, such as a pot. When minor omissions are present, a mesh can be constructed to replace the missing features using, e.g., Autodesk Meshmixer software.

**Figure 13:**
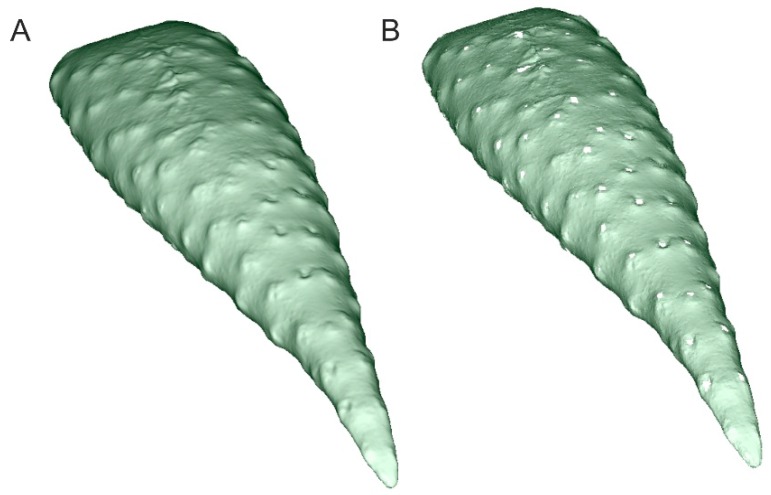
Fragment of an *Aloe marlothii* leaf with visible points where thorns grow: (**A**) Filled areas; (**B**) Holes in place of thorns.

For plants that possess scattered thorns or prickles (plants 1 and 4), the computer model is seldom complete, since the thorns or prickles are visible as empty spaces. The area with empty spots can be calculated; the fragment then needs to be filled and recalculated (Figure 13) to achieve results which are impossible to obtain with standard methods.

## 4. Discussion

Computer models make it possible to determine the area and volume precisely, which lets the researcher calculate the S/V ratio. A method of approximating the S/V ratio by using geometrical primitives exists [44], but computer models can calculate the S/V ratio more accurately. This is especially important for the ecological studies of succulents [20], and such an accurate method has never been used so far.

Three scanners were assessed (DAVID Laserscanner, EXASCAN, and Kinect). The best results for shape and completeness (no missing features, e.g., as in Figure 7 and Figure 10) were obtained with the DAVID Laserscanner. Scan time, which takes approximately 40 min for one plant (with around 35 to 40 scans), is the only disadvantage of working with this device.

A detailed comparison of the DAVID 3D and Kinect scanners has been reported [26]. Phantoms of known geometrical shapes (plane and sphere), and models of plants scanned with the Perceptron v5 coupled to the Romer Infinite 2.0 arm as a “ground truth”, were used to estimate measurements errors. The error for the DAVID 3D device was less then ±1 mm, which is comparable with the manufacturer’s specifications, and is appropriate for phenotyping and parameterization.

Determination of the shortest path in a triangle mesh with the use of Dijkstra’s method has the advantage of correct calculation compared with results from commercial software RapidForm. This method is not suitable for twisted objects such as *Welwitschia mirabilis* leaves, in which the shortest path should be determined as a center line. For such objects it is necessary to use the more sophisticated method presented in Section 2.2.2.

A robust 3D structured light scanning system with a set of five stereo cameras and software focused on whole plant phenotyping has been reported [45]. The use of a motorized table and a multiple-camera set spread over a plant allows us to scan the whole plant in one process. It is not possible with the DAVID Laserscanner which is composed of one camera and one structured light source.

A hand-held scanner (Artec S) has been reported to give a good resolution [5]. However, based on our experience with the EXASCAN device, we feel otherwise. It should, however, be noted that EXASCAN is no longer a state-of-the-art device. Advantages of the DAVID Laserscanner when scanning plants are also reported [24], and they are for Kinect device as well; however, we did not see these. Scanning results obtained by authors with the use of the Kinect sensor (Figure 7B) were not satisfactory. The model is incomplete, which makes it impossible to measure leaf characteristics and to compare with the other scanner. Another approach to Kinect sensor data processing has been described [45]. Segmentation of stems and leaves is performed in 2D depth and image color space instead of 3D scanning.

Non-invasive methods of morphological studies of plants are especially important in studies of plants which are endangered and therefore protected by law. With new methods, it is possible to measure their dimensions and consequently also estimate their age, which has not been possible so far. Exact measures of plants with complicated surfaces and leaf structure are now possible, thanks to phenotyping methods.

## 5. Conclusions

This paper presents the possibilities of using optical scanners to scan and prepare computer models of plants for measurement of their morphological parameters.

Also presented is the analysis of changes in measurement accuracy depending on the triangular mesh resolution in order to assess which resolution yields sufficient measurement accuracy with a reasonable model complexity.

Our work resulted in the development of an original method for leaf length estimation along the line in the middle of the leaf. The method will be used in a study of the *Welwitschia mirabilis* specimens from Adam Mickiewicz University Botanical Garden in Poznan. It can also be used for other plants with a contorted leaf shape. In the future, such models can be used for ecological studies as well as for creating online applications for authorities involved in plant protection.

## Figures and Tables

**Table 1:** List of plant specimens used in the study.

Family	Species	AMU BG Accession Number	CITES Appen.	Ecological Form
Aloaceae	*Aloe marlothii* A. Berger	I_I004_002_0000_6010_4570	II	leaf succulent
Apocynaceae	*Pachypodium lamerei* Drake	I_I004_002_0000_6997_3368	II	stem succulent
Cactaceae	*Astrophytum capricorne* (A. Dietr.) Britton et Rose	I_I002_003_0000_6996_4580	II	stem succulent
Cactaceae	*Astrophytum myriostigma* Lem.	I_I002_003_0000_6996_4581	II	stem succulent
Cactaceae	*Echinopsis leucantha* (Gillies ex Salm-Dyck) Walp.	I_I002_003_0000_6996_4571	II	stem succulent
Cactaceae	*Leuchtenbergia principis* Hook.	I_I002_003_0000_6997_4590	II	stem succulent
Cactaceae	*Mammillaria magnimamma* Haw.	I_I002_003_0000_6999_4573	II	stem succulent
Cactaceae	*Pilosocereus pachycladus* F. Ritter	I_I002_003_0000_6998_4572	II	stem succulent
Welwitschiaceae	*Welwitschia mirabilis* Hook.f.	I_I004_001_0000_6998_4560	I	true xerophyte

**Table 2:** Results of calculating the area, volume, and the distances between points depending on the resolution of the triangular mesh used in various models.

Model Number	1	2	3	4	5	6	7
**Mean distance between vertices [mm]**	**3.97**	**2.03**	**1.09**	**0.56**	**0.28**	**0.20**	**0.14**
Number of points	859	3442	12,013	48,159	191,352	370,824	713,645
Number of triangles	1664	6852	24,022	96,314	382,700	741,632	1,427,286
Area [mm^2^]	11,314	11,919	12,100	12,175	12,206	12,209	12,216
Error %	7.38	2.43	0.95	0.34	0.08	0.06	0.00
Volume [mm^3^]	52,463	55,364	55,993	56,167	56,206	56,172	56,174
Error %	6.61	1.44	0.32	0.01	0.06	0.00	0.00
Distance–triangular mesh [mm]	179.7	184.5	181.7	183.8	183.8	183.1	183.5
Error %	2.10	0.53	0.99	0.12	0.16	0.23	0.00
Distance–segment projection [mm]	169.3	175.7	176.2	176.0	176.4	177.0	177.2
Error %	6.13	5.01	3.17	4.39	4.21	3.45	3.60
S/V ratio	0.22	0.22	0.22	0.22	0.22	0.22	0.22
